# Auxetic Biomedical Metamaterials for Orthopedic Surgery Applications: A Comprehensive Review

**DOI:** 10.1111/os.14142

**Published:** 2024-07-03

**Authors:** Minghao Sun, Xin Hu, Leilei Tian, Xiao Yang, Li Min

**Affiliations:** ^1^ Department of Orthopedic Surgery and Orthopedic Research Institute West China Hospital, Sichuan University Chengdu China; ^2^ Model Worker and Craftsman Talent Innovation Workshop of Sichuan Province Chengdu China; ^3^ Department of Anesthesiology West China Hospital, Sichuan University/West China School of Nursing, Sichuan University Chengdu China; ^4^ National Engineering Research Center for Biomaterials Sichuan University Chengdu China; ^5^ Provincial Engineering Research Center for Biomaterials Genome of Sichuan Sichuan University Chengdu China

**Keywords:** Auxetic biomedical metamaterials, Negative Poisson's ratio, Orthopedics, Surgery

## Abstract

Poisson's ratio in auxetic materials shifts from typically positive to negative, causing lateral expansion during axial tension. This scale‐independent characteristic, originating from tailored architectures, exhibits specific physical properties, including energy adsorption, shear resistance, and fracture resistance. These metamaterials demonstrate exotic mechanical properties with potential applications in several engineering fields, but biomedical applications seem to be one of the most relevant, with an increasing number of articles published in recent years, which present opportunities ranging from cellular repair to organ reconstruction with outstanding mechanical performance, mechanical conduction, and biological activity compared with traditional biomedical metamaterials. Therefore, focusing on understanding the potential of these structures and promoting theoretical and experimental investigations into the benefits of their unique mechanical properties is necessary for achieving high‐performance biomedical applications. Considering the demand for advanced biomaterial implants in surgical technology and the profound advancement of additive manufacturing technology that are particularly relevant to fabricating complex and customizable auxetic mechanical metamaterials, this review focuses on the fundamental geometric configuration and unique physical properties of negative Poisson's ratio materials, then categorizes and summarizes auxetic material applications across some surgical departments, revealing efficacy in joint surgery, spinal surgery, trauma surgery, and sports medicine contexts. Additionally, it emphasizes the substantial potential of auxetic materials as innovative biomedical solutions in orthopedics and demonstrates the significant potential for comprehensive surgical application in the future.

## Graphical abstract

Auxetic biomedical metamaterials for joint surgery, spinal surgery, trauma surgery, and sports medicine applications.

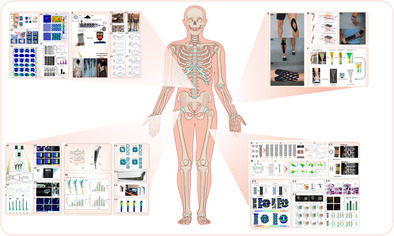

## Introduction

Surgical technology advancements demand superior performance from novel biomaterial implants. Exploring or refining materials is crucial not only to achieve this demand but also to provide more dependable solutions for future surgical procedures.^1^ However, the mechanical properties of natural materials demonstrate a specific/limited range, which limits the design space and practical application of natural materials.^2^ A clear example is Poisson's ratio. Therefore, developing materials that exhibit arbitrarily selected mechanical and physical properties is an urgent need. One illustrative case is mechanical metamaterials—materials that are meticulously engineered with interconnected building blocks, demonstrating unconventional physical characteristics and functionalities rarely found in nature.^3^, ^4^, ^5^, ^6^, ^7^, ^8^ It was originally used in the context of optics and electromagnetism,^4^, ^5^, ^6^, ^7^, ^8^ but today, it refers to all materials engineered to demonstrate novel properties not usually found in nature.^3^ The unusual mechanical properties include low density coupled with high strength,^9^, ^10^ the ability to regain shape,^11^ programmability,^12^ and negative Poisson's ratio (NPR).^13^ The Poisson's ratio (*ν*) of material measures the ratio of transverse strain (*εT*) to longitudinal strain (*εL*) during uniaxial tension or compression, expressed by the equation: *ν* = −*εT*/*εL*.^3^ Among them, those with an NPR have sparked significant interest due to their improved mechanical properties originating from counterintuitive deformation behavior.^14^

Most materials demonstrate a positive Poisson's ratio (PPR) in nature, causing lateral contraction under axial stretching.^1^ However, materials with an NPR, also known as the auxetic effect, undergo lateral expansion during axial tension.^15^ The Poisson's ratio of biomaterials presents several distinctive characteristics. Poisson's ratio in isotropic materials demonstrates direction dependence in line with classical elasticity theory.^16^, ^17^ Importantly, Poisson's ratio for anisotropic materials is contingent on the stretch's direction.^18^ In particular, from a biomaterial perspective, organs, such as the lung, heart, and bladder, exhibit inherent anisotropy,^19^, ^20^, ^21^, ^22^ and surgically restoring these organs poses a challenge, necessitating the development of effective biologically‐based reconstruction therapeutics that can accommodate the intricate mechanics of dynamic organs. Fortunately, anisotropic characteristics within auxetic architectures were induced by modifying lattice design in various directions, allowing biomimicry of organ mechanics.^20^ NPR is a scale‐independent property, indicating that the expansion upon stretching was achieved not only at the macroscopic level but even at the microscopic molecular level.^23^ This unique property is not solely associated with the intrinsic or bulk properties of the selected material but also originates from its precisely tailored architectures.^2^, ^24^, ^25^, ^26^ These physical characteristics of the Poisson's ratio in biomaterials greatly promote the application scope of NPR biomedical materials^3^ and heightened mechanical performance, including improved impact resistance, fracture toughness, isotropy, variable permeability, and energy‐absorbing damping,^27^, ^28^, ^29^, ^30^, ^31^ and broaden the boundaries of surgical techniques when implants adopt an NPR structure, ultimately benefiting a diverse patient population. This enables such materials to effectively meet various surgical needs at different levels, ranging from microscopic cellular tissue repair to macroscopic organ reconstruction.^19^, ^20^, ^21^, ^32^, ^33^

The history of auxetic materials dates back to the 1980s. Since Lakes converted open‐cell polymeric foam into an auxetic foam with a Poisson's ratio of −0.7 in 1987,^34^ auxetic metamaterials have attracted considerable attention for their augmented mechanical properties.^14^ To date, various auxetic materials and structures have been discovered, fabricated, or synthesized, ranging from the macroscopic down to the molecular levels, including auxetic polymeric materials, such as foam, fiber,^35^, ^36^ or even composite, including polyacrylic acid, polyurethane, thermoplastic polyurethane (TPU), polylactic acid, polypropylene, polyethylene glycol, poly (ε‐caprolactone) (PCL), poly (D, L‐lactic‐co‐glycolic acid), etc.^37^, ^38^, ^39^, ^40^, ^41^ They discovered multifaceted applications across diverse domains, including sensors,^42^, ^43^ energy,^44^ textiles,^45^, ^46^, ^47^ and biomedical applications.^44^, ^48^, ^49^ Many exhibit characteristics of NPR, such as bone tissue, muscles, skin tissue, and ligament tissue, for the biological tissues constituting the human body.^49^, ^50^, ^51^, ^52^, ^53^ However, traditional biomedical materials frequently overlook the crucial physical property of Poisson's ratio, preventing fundamental biological simulation reconstruction.^2^ Notably, researchers have focused on the significant potential of auxetic materials in the biomedical field, particularly in clinical surgery, over the past few decades.^3^ Its applications not only span beyond macroscopic trauma repair and structural reconstruction across various human organ systems but also delve into the microscopic realm to foster cellular repair and regeneration. In particular, musculoskeletal applications of artificial intervertebral discs,^38^ porous vertebral pedicle screws,^54^ and femoral stem prostheses,^55^, ^56^, ^57^ cardiovascular applications with auxetic structural coronary stents^58^, ^59^ and cardiac repair patches,^19^ respiratory use of auxetic lung patches,^20^ digestive application of auxetic meshes that prevent pelvic organ prolapse,^21^ dermatology developments, such as three‐dimensional (3D)‐printed wound dressings and skin sensors,^60^ and microscopic applications with auxetic scaffolds that improve osteoblast proliferation for bone tissue regeneration^32^, ^33^—all demonstrate satisfactory clinical efficacy.

At present, the structure manufacturing methods with NPR include traditional thermo‐mechanical and molding processes, which limits the precise tailoring of highly ordered ideal architectures and makes it difficult to achieve large‐scale commercial production. Fortunately, the profound advancement of additive manufacturing (AM) technology, also known as 3D printing technology, allows the high‐precision fabrication of the internal microstructure of biomaterials, enabling flexible adjustments to the material's geometric and topological porous characteristics.^61^, ^62^ This includes stereolithography method,^63^ digital light processing method,^64^ fused deposition modeling method,^65^ selective laser sintering method,^66^ melt electrowriting (MEW) method,^67^ multiphoton lithography method.^68^ This breakthrough facilitates the development of unprecedented meta‐biomaterials that combine outstanding mechanical performance, mechanical conduction, and biological activity.^9^, ^10^, ^11^, ^69^, ^70^ Our study comprehensively reviews and analyzes existing literature on auxetic material integration. It further categorizes and summarizes the current cutting‐edge applications of these materials in orthopedics. Auxetic materials demonstrate a potential as prospective biomedical materials, especially within the orthopedics domain. This review aims to provide a cohesive overview, emphasizing the considerable potential applications of auxetic materials in orthopedics.

## Literature Search Methodology

### Inclusion and Exclusion Criteria

The inclusion criteria were defined as follows: (i) study type—original articles, clinical articles, review articles, guidelines, case reports, surgical techniques; (ii) the literature related to orthopedic surgery, including joint surgery, spinal surgery, trauma surgery, and sports medicine; and (iii) the literature related to the basic fundamental geometric configuration of auxetic metamaterials. The literature related to cell tissue engineering and literature related to other fields, such as material industry, textile industry and computer electronics industry were excluded.

### Study Retrieval and Keywords

**FIGURE 1:**
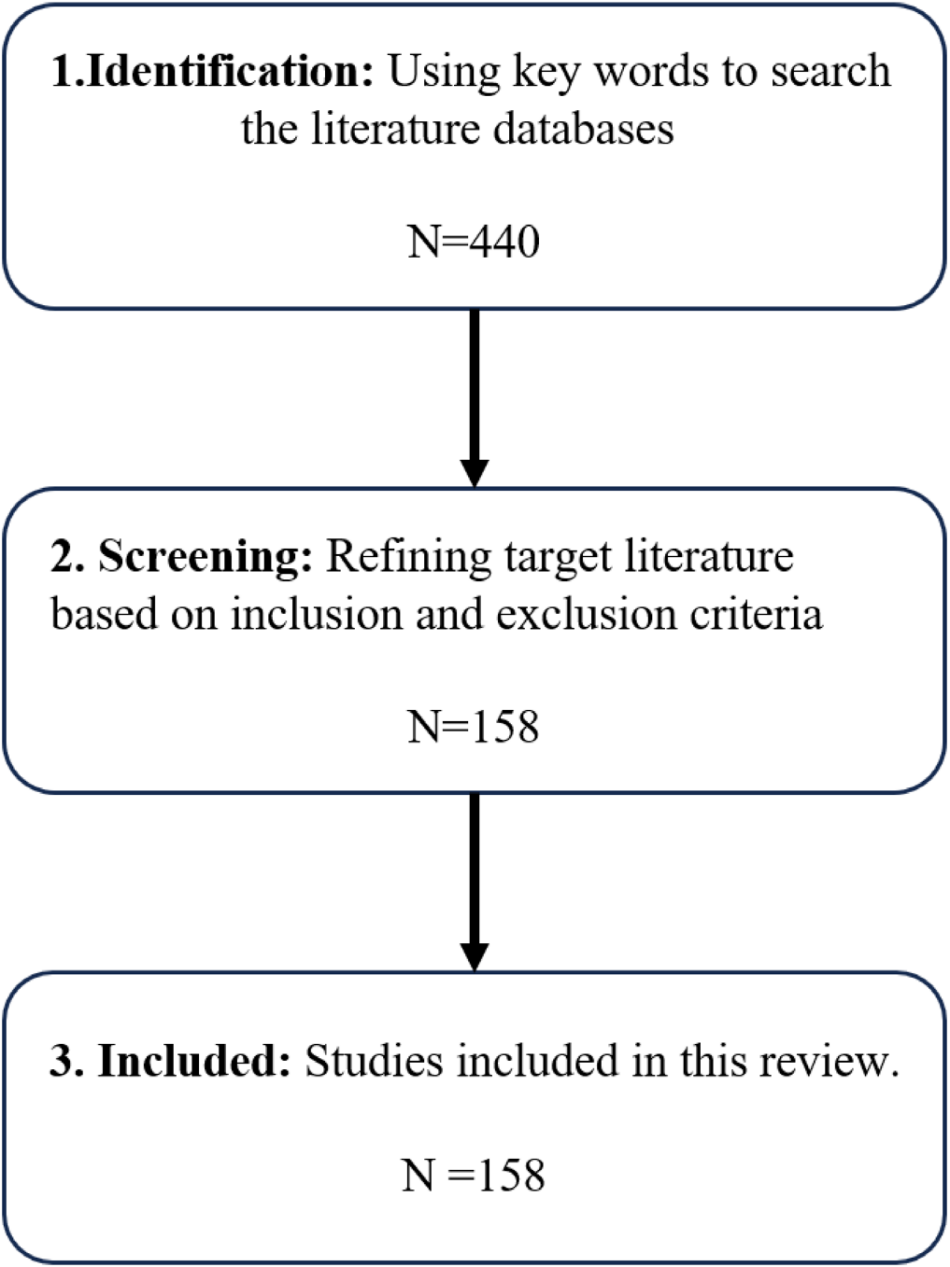
The methodology for literature screening.

A total of 6 databases, including PubMed, Web of Science, Cochrane Library, SCOPUS, CNKI (China National Knowledge Infrastructure), and Wanfang Data Knowledge Service Platform were utilized for study retrieval from database establishment to May 2024. The following keywords and their synonyms were employed for the search: auxetic, negative Poisson's ratio, auxetic biomedical metamaterials; surgery; orthopedics. The retrieval and screening process are illustrated in Figure 1.

## The Fundamental Geometric Configuration of Auxetic Metamaterials

Auxetic structures include three fundamental types: reentrant, chiral, and rotating rigid structures. Varied geometric shapes in auxetic structures yield distinct deformation mechanisms, causing the material's NPR. This has significant consequences for surgical reconstruction.

### Reentrant Structures

**FIGURE 2:**
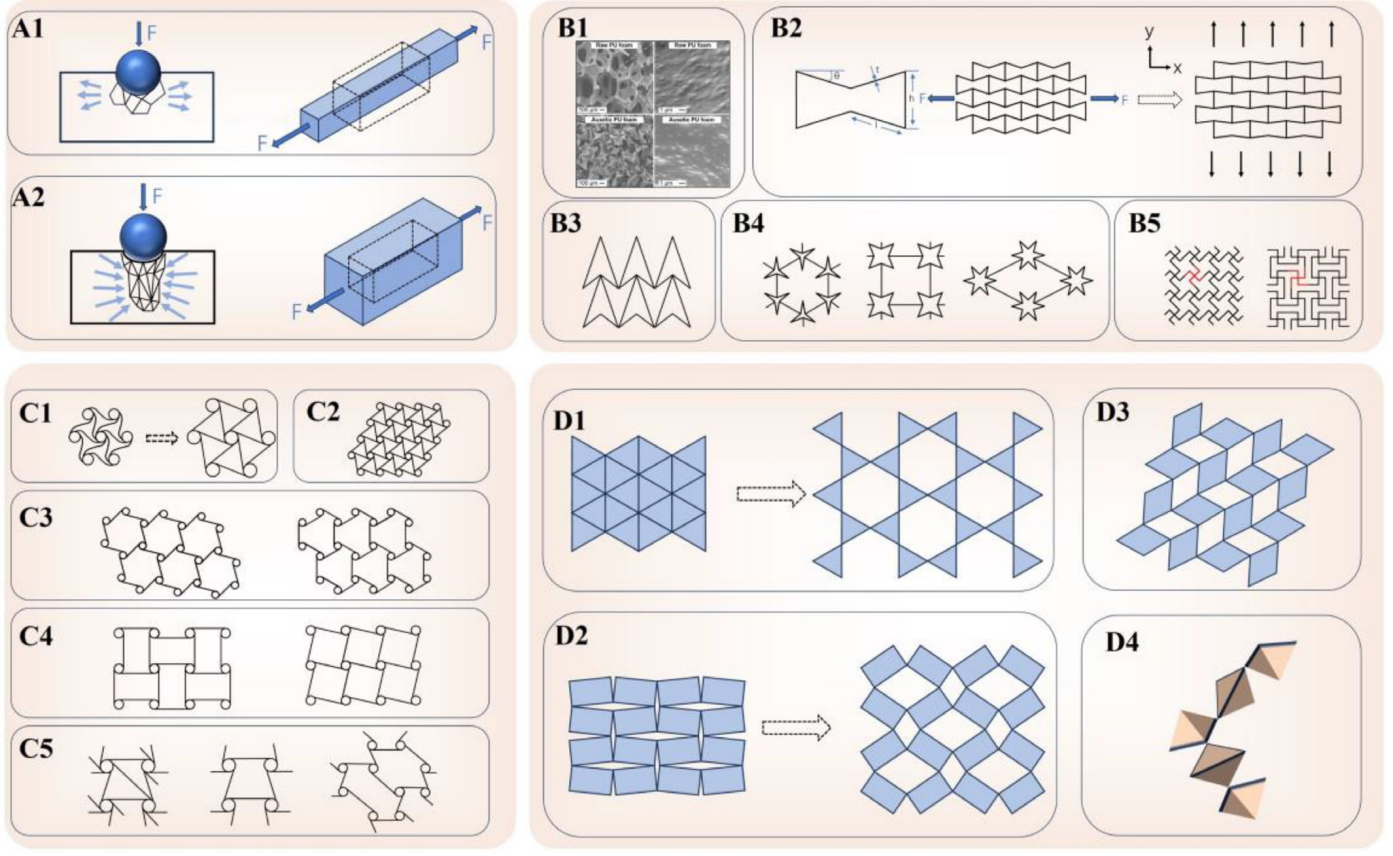
The Figure illustrates the fundamental geometric configuration of auxetic metamaterials. Deformation profile of (A1) non‐auxetic material and (A2) auxetic material. The dashed outline is initially undeformed material. (B1) Scanning electron microscopy images of raw PU foam and auxetic PU foam. (B2) Illustration of auxetic behavior on reentrant honeycomb structures. (B3) Reentrant honeycomb structure of an arrowhead. (B4) Reentrant honeycomb structure of 3‐stars, 4‐stars, and 6‐stars. (B5) Reentrant honeycomb structure of the missing rib foam models (lozenge grid and square grid). (C1) Deformation of a hexachiral unit. (C2) hexachiral structure. (C3) trichiral and anti‐trichiral structures. (C4) tetrachiral and anti‐tetrachiral structures. (C5) meta‐chiral structures. (D1) The rotating rigid structures of triangles. (D2) The rotating rigid structures of rectangles. (D3) The rotating rigid structures of rhombi. (D4) The rotating rigid structures of tetrahedra.

Reentrant, indicating structures directed inward or featuring a negative angle (Figure 2B), undergo deformation primarily through the cell wall realignment (hinging).^62^ In theory, stretching aligns diagonal walls horizontally, causing vertical separation and a stretch expansion effect. Wall deflection and axial deformation (stretching) further contribute to this behavior.^71^, ^72^ Converting two‐dimensional reentrant structures to 3D forms is predominant for crafting materials with an NPR. Techniques, such as electron beam melting (EBM) and direct laser writing, enable customized geometry production on various scales. In particular, Li *et al*. used EBM for an idealized Ti‐6Al‐4V reentrant structure,^73^ and Bückmann *et al*. utilized direct laser writing for similar structures.^74^ Inkjet printing^75^ and dual‐material PolyJet technology^76^ have generated stretchable materials with an NPR.

Typical reentrant structures are observed in foam and honeycomb configurations. Foam structures (Figure 2B1): various conventional foam materials, including thermoplastic foams,^34^ polyurethane foams,^77^ thermoset silicone rubber foams,^35^ and metallic foams based on copper and aluminum,^78^ can be used to produce foams with an NPR. Honeycomb structures (Figure 2B2): In 1982, Gibson *et al*. introduced macroscopic auxetic cellular structures derived from conventional honeycombs represented as 2D reentrant honeycombs.^79^ These provided improved energy absorption, deformation capabilities, and high anisotropy, unlike traditional honeycomb structures with in‐plane isotropy. Reentrant hexagonal honeycomb materials' auxetic effect is influenced by parameters, such as length ratio (h/l), wall thickness, and reentrant angle (θ) of the basic unit.^80^ Studies have revealed that changes in density affect in‐plane Poisson's ratios and Young's moduli, with a wall thickness reduction, altering NPR values and Young's modulus.^81^ Other reentrant geometries have been confirmed to demonstrate the auxetic effect under external loads, apart from the classical reentrant hexagonal honeycomb structure. Examples include the auxetic arrowhead structure (Figure 2B3),^82^, ^83^ stars (Figure 2B4),^84^ lozenge grid, and square grid (Figure 2B5).^85^ The NPR values for the auxetic arrowhead structure range between −0.80 and −0.9296.^82^ The auxetic effect of the star model can be tuned by adjusting the stiffness of hinges and wall thickness under external loads, with the STAR‐6 model demonstrating more potential auxetic behavior compared to the 3‐star and 4‐star models.^84^, ^86^ Both the lozenge and square grids, also known as missing wall foam models,^72^ exhibit in‐plane NPR of approximately −0.43 and −0.699, respectively.^85^

### Chiral Structures

Unlike reentrant structures, chiral structures consistently demonstrate an NPR regardless of specific angles. Straight ligaments (walls) in classical chiral units connect to a central node, which can be circular, rectangular, or nonsuperimposable shape in mirror images.^87^ Walls that connect to a similar side of the central node where the geometry displays reflective symmetry are termed “nonchiral” or “antichiral.”^88^ External tensile or compressive loads in a uniaxial direction induce central node rotation, causing ligaments (walls) to wrap or unwrap, thereby generating an auxetic effect. The structural geometry generates an NPR of approximately −1.^88^ Their ability to “wind” upon compression sustains this ratio over a wide strain range (Figure 2C1).^89^, ^90^ Recognized configurations include trichiral, antitrichiral (Figure 2C3), tetrachiral, antitetrachiral (Figure 2C4), hexachiral (Figure 2C2), and meta‐chiral structures (Figure 2C5).^91^, ^92^

### Rotating Rigid Structures

Unlike the two auxetic structures mentioned above, the ideal rotational structure involves rigid squares connected by hinges. Loaded squares will rotate at the vertices, either expanding or contracting based on the loading type.^93^ Grima and Evans investigated rigid and semi‐rigid square models and revealed an NPR of −1 under external loads.^94^ Therefore, various network types, including triangles (Figure 2D1),^95^ rectangles (Figure 2D2),^23^ rhombi (Figure 2D3),^23^ and tetrahedra (Figure 2D4),^96^ were utilized to achieve auxetic behavior. The NPR value of rotational structures depends on geometric factors and loading direction.^97^ Rotational structures, with highly adjustable NPR values and improved stretchability, hold significant potential in biomedicine.^98^

### Other Structures

In recent years, various other auxetic structures have been reported in addition to the aforementioned structure types. In particular, auxetic gels with 3D porous network structures in hydrogel and aerogel form demonstrate high surface area, high porosity, and good absorptivity, showcasing their potential applications in various fields.^99^ Additionally, other polymer‐based auxetic structures were documented, including bucklicrystal structures,^100^, ^101^ sinusoidal filament networks,^102^, ^103^ and cross‐chiral structures.^104^

There are many factors can affect the property and performance of different types of auxetic structures. The reentrant structures mainly deform through cell wall hinging. This may be improved by diagonal wall thickness reduction (or all walls), reentrant angle θ increase, and cell rib length ratio h/l elevation (up to a certain optimum value).^81^, ^105^, ^106^ The wall thickness directly affects the rigidity of the structure and its overall stiffness. Therefore, Young's modulus decreased as the cell rib thickness decreased and the reentrant angle increased.^81^ The rotation of the nodes and the subsequent flexion of the ligaments guided chiral system deformation. A reduction in the cell rib thickness and an increase in r/R can improve ligament flexion, and, thus, the auxetic effect.^88^, ^107^ The stiffness of these structures has increased with the number of ligaments, their length‐to‐thickness ratio, and the quantity of glue used at the connecting end of the ligament in conventionally obtained structures, thereby making node rotation and ligament flexion more difficult.^88^, ^107^, ^108^, ^109^ The Poisson's ratio was independent of L/r in the antitetrachiral and hexachiral structures, whereas the antitrichiral structure demonstrated a decrease in NPR.^88^ Three‐dimensional chiral auxetic metamaterials demonstrated the same trends,^110^, ^111^ but more research is warranted to investigate the auxetic potential of these three‐dimensional structures, as well as the contradictory information regarding the stiffness of chiral structures versus their antichiral counterparts.^88^, ^112^ Rotating rigid structures deform through the rotation of the rigid units, thereby changing the angles between them. This is directly associated with joint rigidity, which has negatively influenced the auxetic effect.^113^, ^114^ Research shows that reentrant structures outperform the chiral and rotating rigid structures in terms of Poisson's ratio and corresponding stiffness, and most research has been conducted on the reentrant structures, especially the reentrant hexagonal honeycombs.^62^ Chiral and rotating rigid structures were modeled extensively, but experimental data have not yet been widely reported. Therefore, performing a well‐founded comparison between the three basic types of auxetic structures is impossible.^62^

## Auxetic Metamaterials in Clinical Applications in Orthopedics

### Joint Surgery

**FIGURE 3:**
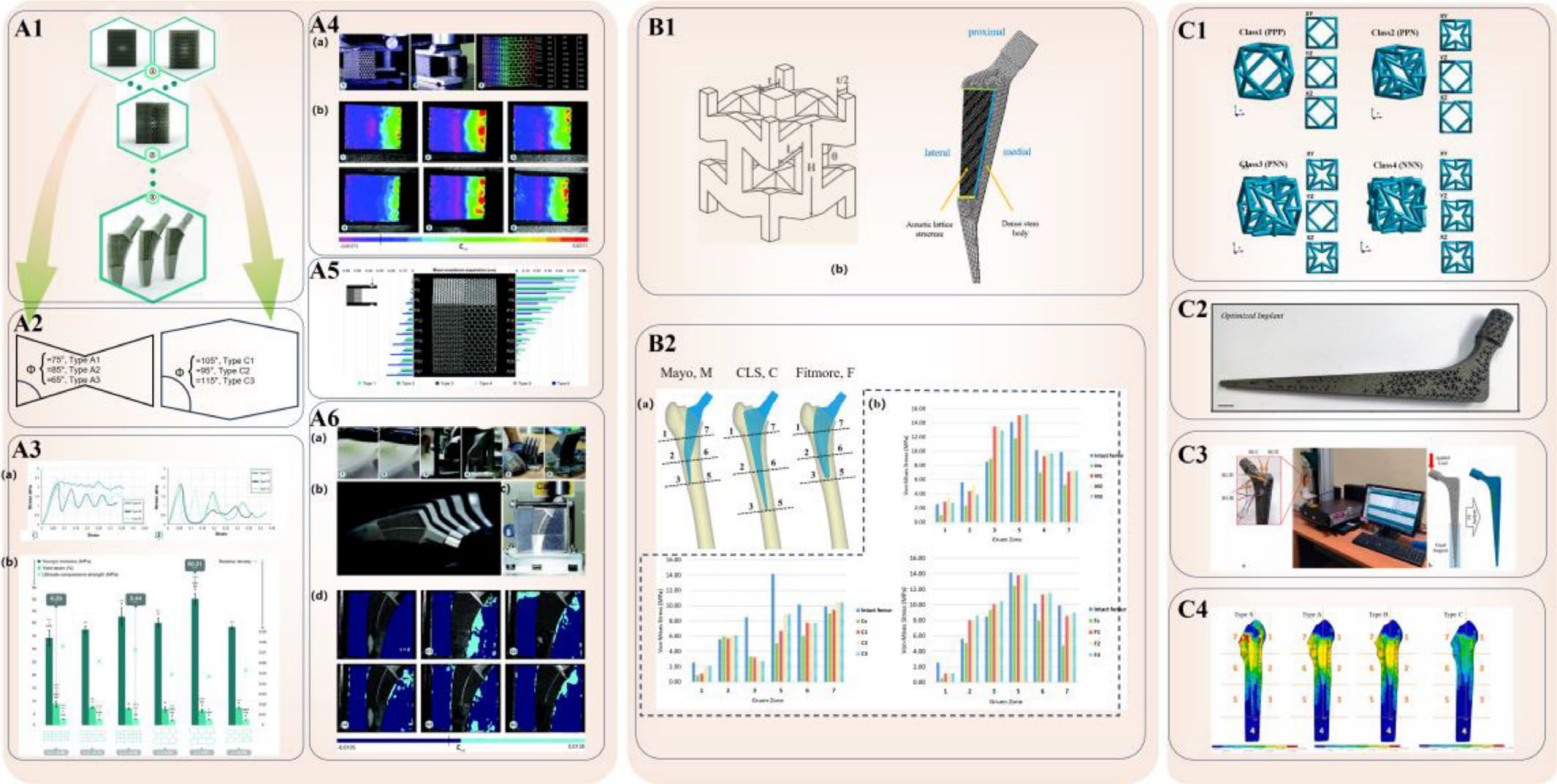
Auxetic biomedical metamaterials for joint surgery applications. (A1) The schematic of the topological designs of auxetic and conventional meta‐biomaterials, hybrid meta‐biomaterials and meta‐implants. (A2) Design of auxetic (left) and conventional (right) meta‐biomaterials with internal angle Ф. (A3) (a) The mean stress–strain curves of (1) auxetic (A1–A3) and conventional (C1–C3) meta‐biomaterials under compression. (b) The mean compressive Young's modulus, yield strain, ultimate compressive strength, relative density, and Poisson's ratio (*ν*) of (from left to right) A1, A2, A3, C1, C2 and C3. Data are expressed as means and error bars indicate 95% confidence intervals. Significant differences are indicated by *p < 0.05 compared with A1, **p < 0.05 compared with A2, ***p < 0.05 compared with A3, compared with C1, compared with C2 and compared with C3 (one‐way analysis of variance). Maximum values are indicated. (A4) (a) Test set‐ups and image processing: (1) off‐axis compression, (2) off‐axis compression with an adherent layer used to improve visualization of strain distribution, (3) points plotted along the borders of a hybrid meta‐biomaterial and the order in which they were numbered according to their position. (b) Horizontal strains in the tape surrounding hybrid meta‐biomaterials types 1–6 at 2 mm displacement. (A5) Mean maximum expansion during off‐axis compression. (A6) (a) Meta‐implants were manufactured with selective laser melting (SLM). (b) Additively manufactured meta‐implants. (c) The test set‐up in which they were loaded including bone‐mimicking materials. (d) Horizontal strains in the bone‐mimicking materials surrounding the meta‐implants at *t* = 0 and *t* = 180 s at 1.5 mm displacement for C1, C2, H1, H2 and H3.^120^ (B1) Design parameters of the auxetic unit cell and hip stem. (B2) Illustration of Gruen zones for each stem type and von Mises stress recorded with M‐type stems, C‐type stems, and F‐type stems respectively.^56^ (C1) Different classes of auxetic structures and their corresponding views in XY, YZ, and ZX planes. (C2) Additively manufactured optimized implant. (C3) Experiment setup for measuring strain under compressive load using four strain gauges attached to the manufactured implant and FE analysis with boundary conditions replicated from the experiment. (C4) Stress shielding on different Gruen zones for type S, type A, type B, and type C implants. Stress shielding was estimated as the percentage decrease of SED on peri‐implant bone relative to intact condition.^55^ Copyright 2023 Elsevier.

Total hip arthroplasty (THA) is the established gold standard for severe hip joint diseases, representing a prevalent and pivotal orthopedic surgical intervention with over 2 million global procedures annually.^115^ THA restores independent ambulation and daily functionality, thereby significantly improving patients' quality of life. However, some cases required revision surgery due to various complications.^116^ Orthopedic implants, predominantly composed of metals and alloys with high hardness, bear a substantial load during THA, inducing stress‐shielding in the surrounding bone.^117^ However, femoral stem aseptic loosening, which is a prevalent cause of implant failure, is closely associated with stress‐shielding.^118^, ^119^ The specialized properties of materials with an NPR provide unique prospects for improving the mechanical optimization of total hip joint implants, presenting a distinctive avenue for investigation in this domain. Liu *et al*. revealed that femoral stems incorporating an auxetic lattice structure experienced lower stress‐shielding (SS) post‐THA when compared to their solid counterparts^56^ (Figure 3B). Interestingly, leveraging auxetic materials and strategically designing diverse regions in the hip stem result in the following unforeseen mechanical optimization:

1. Medial‐to‐lateral differentiated NPR design: In 2017, Kolken *et al*.^120^ innovatively designed and additively manufactured hybrid meta‐biomaterials featuring strategically distributed NPR (medial) and PPR (lateral). These materials were applied in developing hip stems and underwent testing under simulated implantation conditions. Full‐field strain measurements demonstrated that the hybrid meta‐implants applied pressure on both the medial and lateral aspects of the bone, under biomechanical loading. This not only improved the contact between the implant and the bone but also exhibited a potential for prolonging implant lifespan. Furthermore, it decreased the likelihood of bone‐implant interface failure (according to Hoffman's criterion), prevented the ingress of wear particles into the interface space, mitigated stress‐shielding effects, and promoted improved bone ingrowth (Figure 3A). In 2021, the team studied the compression–compression fatigue performance of AM auxetic meta‐biomaterials made from commercially pure titanium (CP‐Ti). The morphology, static mechanical performance, and fatigue behavior of 12 different designs were evaluated. The result reveals that these auxetic meta‐biomaterials demonstrated morphological and mechanical properties that are deemed appropriate for bone‐implant applications (elastic modulus = 66.3–5648 MPa, yield strength = 1.4–46.7 MPa, pore size = 1.3–2.7 mm), and the auxetic structures characterized here are superior to many other nonauxetic meta‐biomaterials made from the same material.^31^ Similarly, Singh *et al*.^57^ modified a solid hip implant by adding auxetic metamaterial on its lateral side to foster a more osseointegration‐friendly proximal‐lateral femur environment, which mitigates bone resorption and wear particle entry issues associated with traditional solid hip implants. In 2023, Liu *et al*. designed a heterostructure with reasonably distributed NPR and PPR structures according to the hip joint's practical application requirements. The results of finite element (FE) analysis reveal that deformation occurs on different sides of the heterogeneous structure that extends outward when a graded strain is applied. Additionally, the heterostructure will press onto the bone on both the medial and lateral sides under biomechanical loading, benefiting from the unique deformation characteristics of the heterostructure. The heterostructure's unique deformation will improve the hip joint's reliability and extend implant longevity.^121^
2. Proximal‐to‐distal NPR design: In 2020, Eldesouky and El‐Hofy^122^ introduced an innovative solution for stress‐shielding issues with a new low‐stiffness femoral hip stem design. The proposed hip stem characterizes a porous auxetic scaffold in the proximal region and a hollow distal end. FE simulations confirmed that this design improves stress and strain distribution in the proximal region, thereby reducing the stress‐shielding effect. Specifically, the stresses in the femur's proximal region are higher with the porous implant compared to a solid implant. Transitioning from a solid to a porous stem increases the maximum von Mises stresses in the femur's medial region from 43.8 to 55.7 MPa. Additionally, the location of the maximum von Mises bone stresses shifts from the distal region to the proximal area, reducing stress‐shielding in the femur's proximal region and potentially minimizing bone absorption.
3. Graded Poisson's ratio distribution design: Ghavidelnia *et al*.^123^ used 3D reentrant auxetic metamaterial to craft a porous femoral hip meta‐implant with a graded Poisson's ratio distribution to boost micromotions at bone‐implant contact surfaces. Numerical results under compressive loading indicated that this hip joint meta‐implant, featuring a well‐designed lattice structure, effectively optimizes stress and strain distributions in both the implant and surrounding bone tissues. Notably, this design innovation successfully resolved the stress‐shielding problem typically associated with solid implants. Additionally, the precision in crafting a graded lattice structure improves implant deformation, thereby fostering a compatible and uniformly distributed micromotion that increases superior bone ingrowth at the bone‐implant interface. Rana *et al*.^55^ undertook updated endeavors, introducing a novel group of orthotropic auxetic structures in a porous Ti6Al4V hip implant, in addition to the aforementioned relatively systematic large‐scale zoning for NPR settings. These structures, exhibiting variable stiffness and Poisson's ratio in different directions, were validated by comparing FE results with experimentally measured properties. The optimized implant significantly reduced average stress‐shielding from 56% to 18%, thereby decreasing the risk of implant loosening and increasing osseointegration‐friendly biomechanics in the surrounding bone (Figure 3C).

### Spinal Surgery

Spinal surgery includes a specialized medical field dedicated to diagnosing and treating spinal and related structural issues. Surgical interventions primarily aimed to restore spinal mechanical integrity and regain lost motor function attributed to degeneration, infections, tumors, trauma, and analogous factors. The effective application of implants with NPR structures in spinal surgery is notable due to their unique deformation mechanism under compression.

**FIGURE 4:**
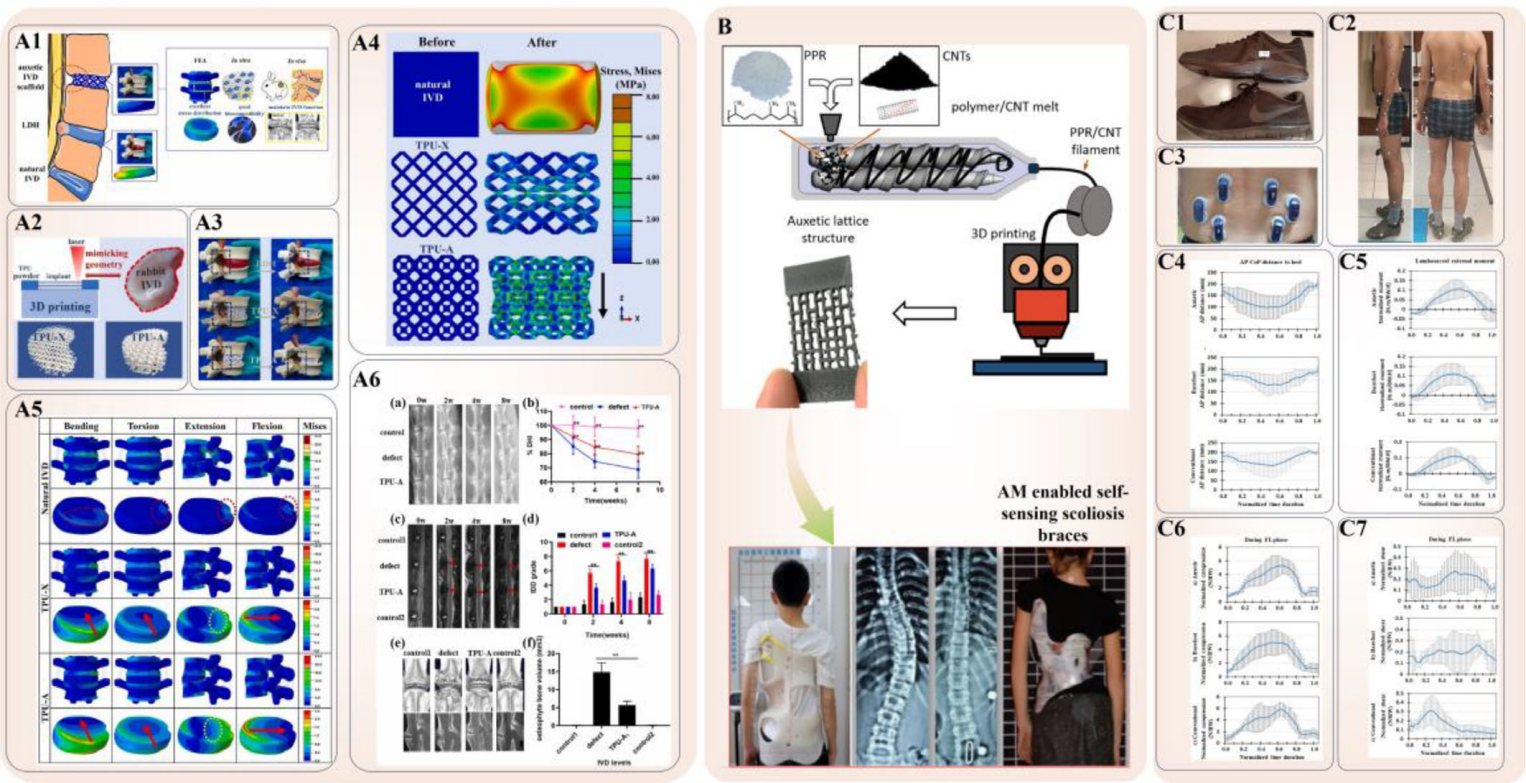
Auxetic biomedical metamaterials for spinal surgery applications. (A1) Schematic showing the auxetic IVD implant subjected to compression and advantages of the auxetic IVD prosthesis. (A2) The SLS fabrication process and printed implants with geometry mimicking the rabbit IVD. (A3) The behavior of TPU‐X and TPU‐A under compression using a commercial LDH model. (A4) The stress and deformation distribution within natural IVD, TPU‐X and TPU‐A under compression. (A5) The equivalent stress distribution and deformation distribution of L4‐L5 lumbar under bending, torsion, extension and flexion conditions. (A6) Radiographic changes of spine after the TPU‐A implantation: (a) X‐ray images and (b) measurements of DHI% changes. (c) MRI images and (d) IDD grading score (modified pfirrmann grading) at different time points after surgery. (e) 3D re constructions of μ‐CT scans of the rabbit lumbar spines and the representative cross‐sections 8 weeks after implantation, (f) quantification of osteophyte bone volumes after different treatment. Error bars represent S.D., and significant differences were assessed with Student's *t*‐test; *p < 0.05, **p < 0.01.^38^ (B) Feedstock filament fabrication (*via* extrusion) and fused filament fabrication (FFF) of polypropylene random copolymer (PPR)/carbon nanotube (CNT) nanocomposites. The 3D printed auxetic lattice structures shown are intended for testing the piezoresistive behavior and development of individually tailored biomedical devices, such as scoliosis braces^127^ Copyright 2022 American Chemical Society. (C1) Conventional running shoe (upper) and Auxetic Nike free RN shoe (lower). (C2) Placement of Vicon markers in the sagittal and frontal planes. (C3) Placement of EMG electrodes on back muscles. (C4) Anterior–posterior (AP) distance between center of pressure (CoP) of ground reaction force (GRF) and heel (mm) (± one standard deviation) during the first landing (FL) phase of DVJ in three footwear conditions. (C5) Mean normalized lumbosacral external moment (± one standard deviation) during the first landing (FL) phase of DVJ in three footwear conditions. (C6) Mean normalized lumbosacral (L5‐S1) compression force for all the participants (± one standard deviation) during the first landing (FL) phase in three footwear conditions. (C7) Mean normalized lumbosacral (L5‐S1) shear force for all the participants (± one standard deviation) during the first landing (FL) phase in three footwear conditions.^128^

A 2023 study led by Jiang *et al*.^38^ exemplified the distinctive use of auxetic properties in spinal surgery. They innovatively designed and 3D printed an artificial intervertebral disc implant featuring a modified “Bucklicrystal” structure using TPU. The implant's NPR significantly contributes to superior energy absorption and stability under compression. FE analysis (FEA) reveals improved stress transfer and attenuation compared to both natural intervertebral discs and conventional 3D implants. Remarkably, the implant's lateral contraction under compression demonstrates the potential for alleviating symptoms associated with lumbar disc herniation. Additionally, *in vitro* studies confirm the excellent biocompatibility of TPU‐A. Further, *in vivo* implantation in a rabbit disc model substantiates its capacity to sustain natural motion segment function and endure practical loading conditions (Figure 4A). Additionally, they designed a reentrant honeycomb structure that was 3D printed into a PCL scaffold with the NPR effect for treating lumbar herniated discs. Afterward, the behaviors of the obtained composite implant under loading conditions were analyzed using a mechanical testing machine and FE simulation. The result revealed that as an annulus fibrosus implant, the NPR scaffold with excellent stiffness compared with PPR could not only sustain axial spine loading but also resist nucleus pulposus swelling and increase uniform stress diffusion during nucleus pulposus swelling and contraction. The *in vitro* biological analysis illustrated that this scaffold demonstrated excellent biocompatibility and cell adhesion after PPy coating. Additionally, *in vivo* implantation in the rat disc model confirmed that the scaffold exhibited better radiographic changes and demonstrated the potential to reestablish the function of native spine motion segments and alleviate intervertebral disc degeneration.^124^ Similarly, related biomechanical research on artificial intervertebral discs with an NPR, as conducted by scholars, such as Baker,^125^ has focused on FEA of the L4–L5 motion segment. Their investigation aimed to identify the effect of changing Poisson's ratio of the disc on stresses, range of motion, and displacement of the intervertebral disc. These analyses reveal that an NPR artificial intervertebral disc undergoes stress and range of motion similar to a natural intervertebral disc. Importantly, this effect decreases transverse motion, thereby preventing the impingement of nerves by the intervertebral disc.

In 2023, Lvov *et al*.^126^ proposed an approach for adapting a commercially available interbody cage for auxetic metamaterial and honeycomb structure using standard operations in computer‐aided design software. The mechanical properties of experimental prototypes of Ti‐6Al‐4 V cages made by selective laser melting using computer modeling (FEA), and static and low‐cycle fatigue compression tests (up to 3500 cycles) were then characterized. The result revealed that auxetic metamaterials demonstrated higher static compressive strength and fatigue resistance than traditional honeycomb structures. The Young modulus of the auxetic‐based interbody cage is 6.68 ± 0.28 GPa and comparable to the elastic modulus of human cortical bone. Importantly, the auxetic‐based cage is not destroyed after 3500 cycles under 14‐kN load with residual deformations of ≤ 1% (0.21 ± 0.10 mm of displacements).

Smart wearable devices discovered effective applications in spinal surgery, in addition to surgical reconstruction based on NPR intervertebral disc implants. In 2022, Verma *et al*.^127^ introduced a novel approach that involves carbon nanotube‐reinforced polypropylene random copolymer nanocomposites for additive manufacturing using fused filament fabrication (FFF). These nanocomposites enable the fabrication of lattice structures with self‐sensing capabilities, showcasing NPR characteristics, particularly in strain‐ and damage‐sensing under tension. In particular, the application of these materials in creating self‐sensing orthopedic bracing showed potential for treating complex three‐dimensional deformities in adolescent patients with scoliosis. Such bracing provided the capability to sense and precisely adjust stiffness in various directions, thereby optimizing the timing and method of brace adjustments (Figure 4B). Additionally, In 2022, Dehaghani *et al*.^128^ conducted a study investigating the influence of auxetic shoes on spinal biomechanics in contrast to barefoot and conventional shoe conditions. This investigation encompassed gait and drop vertical jump activities, using a combined *in vivo* and musculoskeletal modeling approach. The results reveal the potential use of auxetic shoes in alleviating lumbar spine load during high‐impact activities, such as vertical jumps, thereby potentially decreasing the musculoskeletal risk of injuries during such movements (Figure 4C).

### Trauma Surgery

Injuries are expected to significantly cause death and disability globally, affecting diverse populations.^129^, ^130^, ^131^ Fractures, including those of long bones, the pelvis, and the spine, are increasingly recognized as the primary type of trauma.^132^ However, the conventional use of bone screws and plates for fixation is associated with clinical challenges, such as screw loosening, migration, and fractures, requiring additional surgeries.^133^, ^134^, ^135^, ^136^ A significant factor contributing to mechanical failures is the lack of an effective stress transmission mechanism after the internal fixation of fractures, making it challenging to achieve the long‐term stability of fixation.^134^, ^135^, ^137^, ^138^ Therefore, integrating auxetic structures into the bone plate/screw design appeared as a promising solution to alleviate stress‐shielding, facilitate intraoperative bending, and bolster the bone healing process.

Vijayavenkataraman *et al*.^139^ introduced an innovative bone plate design featuring auxetic structures. Incorporating the auxetic structure not only reduces stiffness, which is expected to diminish the stress‐shielding effect, but also acts as a deformable segment, which facilitates effective intraoperative bending for alignment during surgery. FEA studies highlight a substantial decrease in maximum stress in bone plates that incorporate auxetic structures, indicating a potential stress‐shielding effect reduction and bone healing process improvement. Additionally, the researchers conducted a comparative analysis between the two studied auxetic structures. The reentrant honeycomb structure demonstrated a more pronounced NPR behavior compared to the missing wall structure. Bone plates that incorporate the reentrant honeycomb structure demonstrate superior bending strength, lower bending stiffness, and smoother bending contours compared to traditional designs. Furthermore, they revealed resilience in fatigue testing, enduring 100,000 cycles, in stark contrast to bone plate failure with missing wall structures before reaching 10,000 cycles.

**FIGURE 5:**
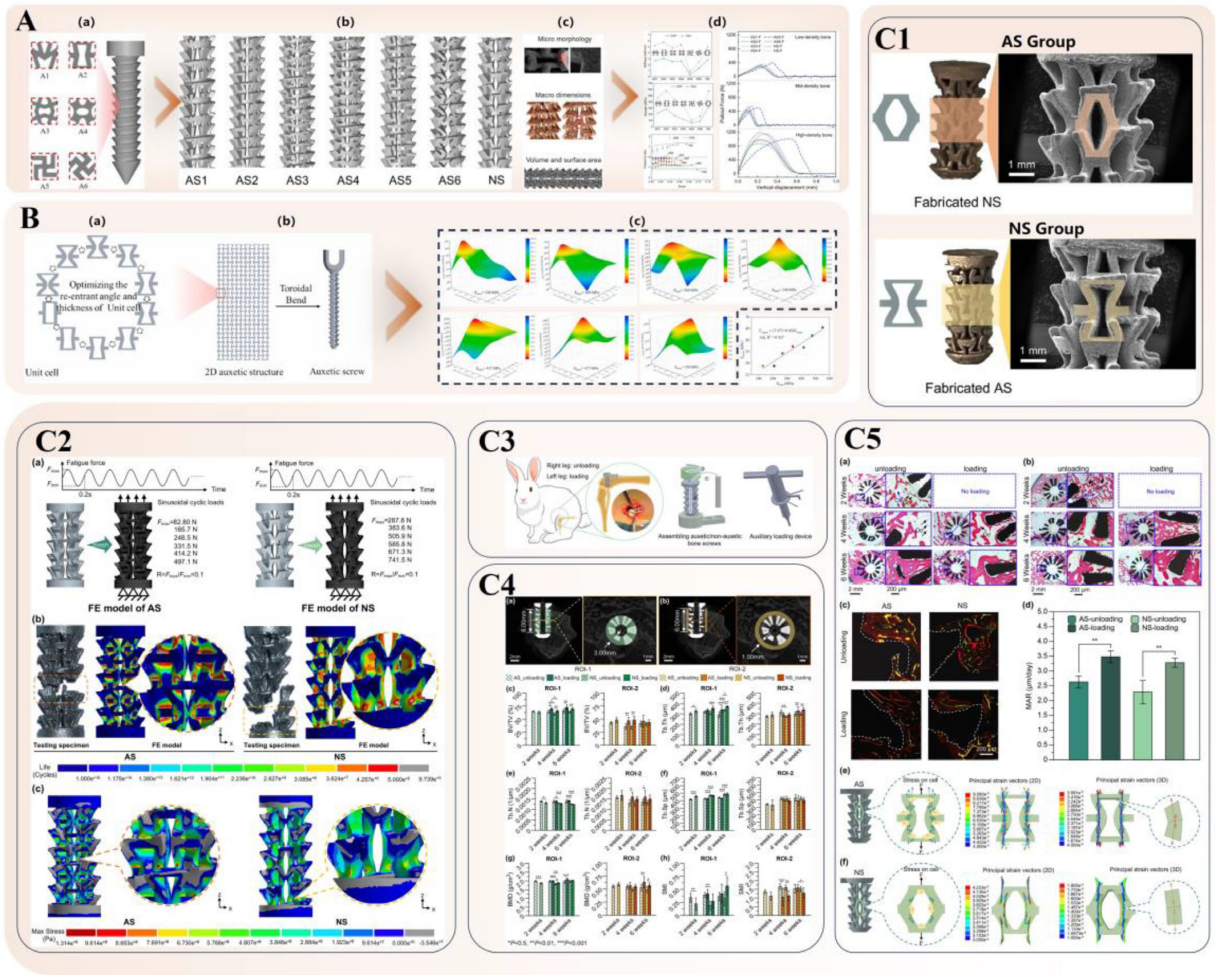
Auxetic biomedical metamaterials for trauma surgery applications. (A) (a) Auxetic unit cells (A1–A6). (b) Designed screw bodies: AS1, AS2, AS3, AS4, AS5, AS6, and NS. (c) The morphological and mechanical characterization of the screws. (d) Mechanical properties of bone screw.^140^ (B) (a) The auxetic unit cells. (b) The auxetic pedicle screw consisted of auxetic unit cells. (c) The maximum pullout forces of bone screws with different design parameters in different types of bone, and the linear fitting of the E_screw_ of the screw with the maximum pullout force and the E_bone_ of the surrounding bone^141^ Copyright 2021 Elsevier. (C1) Surfacial morphologies of fabricated AS and NS. (C2) Fatigue numerical simulation results of AS and NS under different nominal stress levels. (a) Geometries and boundary conditions of the FE models. (b) Fatigue life distributions of AS and NS under the maximum fatigue stress level. (c) Maximum stress distributions of AS and NS under the maximum fatigue stress level. (C3) Schematic illustration of *in vivo* animal implantation. (C4) Regions of interest (ROIs) in micro‐CT reconstruction and quantitative osseointegration parameters of AS and NS without loading/with dynamic tensile loading. (a) ROI‐1: the internal region of bone screw. (b) ROI‐2: the peripheral region of bone screw. (c) Comparison of bone volume fraction (BV/TV) in ROI‐1 and ROI‐2 among different subgroups. (d) Comparison of trabecular thickness (Tb.Th) in ROI‐1 and ROI‐2 among different subgroups. (e) Comparison of trabecular number (Tb.N) in ROI‐1 and ROI‐2 among different subgroups. (f) Comparison of trabecular separation (Tb.Sp) in ROI‐1 and ROI‐2 among different subgroups. (g) Comparison of bone mineral density (BMD) in ROI‐1 and ROI‐2 among different subgroups. (h) Comparison of structural pattern index (SMI) in ROI‐1 and ROI‐2 among different subgroups. (C5) Histological and biomechanical analysis of osseointegration for AS and NS without loading/with dynamic tensile loading. (a) Histological stained sections of AS in different subgroups (green arrows: osteoblasts (Ob); black arrows: osteoclasts (Oc)). (b) Histological stained sections of AS in different subgroups (Green arrows: osteoblasts (Ob); black arrows: osteoclasts (Oc)). (c) Merged results of fluorescent‐labeled sections of AS group at different periods after implantation (Region circled by white dotted line: bone screw; green fluorescent band: newly formed bone tissues at 2 weeks after implantation; Yellow fluorescent band: newly formed bone tissues at 4 weeks after implantation; Red fluorescent band: newly formed bone tissues at 6 weeks after implantation). (d) Comparison of MAR between AS and NS without/with dynamic tensile loading after 6 weeks' implantation. (e, f) Biomechanical analysis based on FEA^54^ Copyright 2023 Elsevier.

In the realm of auxetic screws, Yao *et al*. pioneered the concept of NPR structures for screws in 2020.^140^ They introduced six types of auxetic unit cells into the design of bone screws using the selected laser melting 3D printing method, with a typical nonauxetic unit serving as a comparison. The tensile stiffness, strength, and Poisson's ratio of the designed unit cells and screws were evaluated through tensile tests and FEA. The results revealed that changes in the type of auxetic structure significantly affected the mechanical properties of the screw, especially its functional aspects. Auxetic bone screws that incorporate reentrant structures (A1 and A2) and chiral structures (A3 and A4) demonstrated superior tensile stiffness and strength, whereas those composed of reentrant structures (A1 and A2) and rotating structures (A5 and A6) exhibited improved auxetic performance. Subsequently, a comparison based on FEA was conducted among auxetic bone screws (ASs), nonauxetic bone screws (NSs), and hollow bone screws (HS, used in clinics) regarding pullout fixation strength. The results indicated that AS1–AS6 demonstrated higher pullout forces than NS and HS across all three bone‐density groups. Specifically, the maximum pullout forces in low‐, mid‐, and high‐density bones were observed in AS5 (399.39 N), AS6 (561.07 N), and AS2 (1185.93 N), respectively (Figure 5A).

Building on the previous research, the team introduced an innovative auxetic structures‐based pedicle screw in 2021,^141^ enabling radial expansion under tension for improved bone screw fixation. FE models were used to analyze mechanical properties with varying reentrant angles and thicknesses, specifically addressing screw loosening in spinal fusion surgery. Results revealed that the auxetic deformation improved fixation, which increased pullout force by 6.29%–14.46% compared to NS (Figure 5B). In 2023,^54^ following prior research, the team implanted AS and NS in a rabbit femoral condyle model while assessing *in vivo* osseointegration. AS, with its structural advantages, significantly increased bone ingrowth compared to NS, thereby contributing to preferable long‐term stability. Both AS and NS, under dynamic tensile loading, demonstrated improved *in vivo* osseointegration, with AS favorably affecting osteoblast proliferation and differentiation. AS showed lower *in vitro* fatigue strength than NS, but its improved osseointegration compensated for this limitation, ensuring sufficient long‐term stability postimplantation. This study confirms that AS provides both initial and long‐term stability and biocompatibility, thereby providing efficient screw‐bone fixation. Addressing solid bone screw limitations, it fosters surgical success and shows potential in clinical applications (Figure 5C).

Similarly, in 2024, Barnett *et al*.^142^ investigated the effect of four auxetic structures (the reentrant, rotating squares, missing rib, and tetrachiral structures) on the pullout performance of a novel unthreaded bone fastener through experiments and numerical simulations. Results indicated improved pullout resistance in all the auxetic structures investigated relative to a nonauxetic control sample. The rotating squares structure demonstrated the maximum pullout force of 99.5 N, which is 2.5 times using bone analog material that was achieved by the control. Additionally, it achieved high pullout resistance with a low insertion force that improves ease of installation with a pullout to push‐in force ratio of 33.7. Further, the effect of increased diametral interference was investigated, and the reentrant structure demonstrated superiority, with pullout resistance improved by 342%.

### Sports Medicine

**FIGURE 6:**
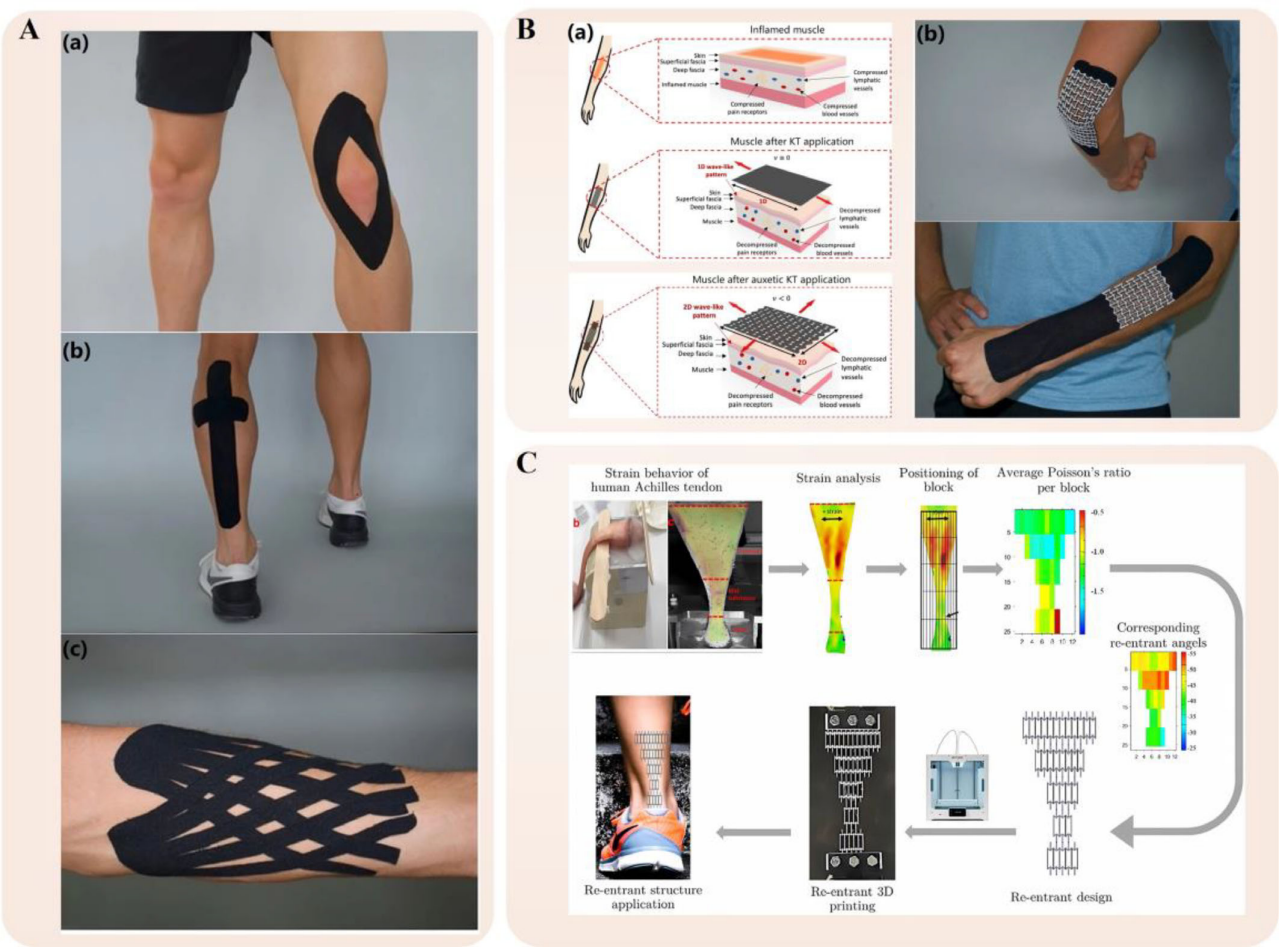
Auxetic biomedical metamaterials for sports medicine applications. (A) Examples of KT applications on the (a) knee, the (b) calf and the (c) forearm.^150^ (B) (a) The compression on the skin, fascia, and muscle due to an injury, and muscle after KT and auxetic KT application. (b) The auxetic KT for elbow pain and forearm muscle tenseness.^150^ (C) The steps of analyzing, designing and manufacturing of novel KT Achilles tendon.^151^

Sports injuries disrupt the regular physical activities of both professional athletes and recreational enthusiasts, thereby negatively affecting their overall quality of life and habits. Research indicates that 65% of young athletes experience sports‐related injuries annually.^143^ Among various injuries, Achilles tendon injury is one of the most prevalent tendon injuries among athletes and the general public.^144^ The current approach for fast recovery involves a combination of therapeutic interventions and exercises.^145^, ^146^ A predominant adjunctive treatment in the rehabilitation process is the use of kinesiology tape (KT), which is a flexible and adhesive tape. KT, invented by Kenzo Kase in the 1970s, is valued in treating sports injuries due to its ability to improve blood flow in the skin.^147^ Additionally, the use of KT has been associated with improved lymphatic fluid circulation, which is primarily composed of water and proteins, thereby helping in swelling regulation (Figure 6A).^148^, ^149^

In 2015, Gatt *et al*.^50^ conducted biomechanical experiments on vertebrate cadavers, investigating tendons from pigs, sheep, and humans. Results indicated an NPR in these tendons. Further, *in vivo* experiments on human subjects revealed that the left leg Achilles tendon exhibited an NPR during dorsiflexion, confirmed through magnetic resonance imaging with a Gyroscan (Philips, 1.5 T, Amsterdam, the Netherlands) and a dedicated ankle/foot quadrature coil. In 2022, Meeusen *et al*.^150^ introduced an innovative KT that features auxetic structures. They initially crafted the auxetic design by laser‐cutting existing KT and later used TPU material and FFF technology for 3D printing additional structures onto the laser‐cut tape. This advancement was evaluated in two common applications: addressing general elbow pain caused by overuse or stretching and treating tenseness in forearm muscles. Results indicated that the auxetic KT, with its synclastic curvature, better conformed to the elbow's shape compared to traditional KT, which simplifies the application process. It demonstrated superior potential in both scenarios, which reduce application steps and provide personalized comfort through a free range of motion and adjustable tape rigidity based on the user's skin and injury type (Figure 6B). Additionally, Hedayati *et al*.^151^ advanced previous research by precisely identifying the Poisson's ratio across various Achilles tendon regions. They innovatively used 3D printing technology, utilizing TPU material, to develop a novel auxetic Kinesio tape. This tape featured a nonuniform distribution of reentrant unit cells, strategically designed to closely match the mechanical properties, including strain distributions and Poisson's ratios, of the Achilles tendon. Numerical and experimental results revealed that the newly designed KT effectively replicated the deformation characteristics of the Achilles tendon in both longitudinal and transverse directions. Notably, the reentrant structure of the tape proved instrumental in ensuring efficient skin adhesion above the Achilles tendon. This study not only demonstrates the tape's potential in recovery applications but also provides a theoretical foundation for future developments in 3D bioprinting of Achilles tendon implants (Figure 6C).

In the realm of KT, Kwan^152^ developed a stretchable silicone band with an auxetic structure to correct the hallux valgus in 2024. The reentrant auxetic structure is used to improve the corrective band fit. This study demonstrates that the auxetic property allows the auxetic structure to expand and shrink in the transverse direction above and below the neutral plane during bending, and contributes to synclastic curvature, which allows the creation of a dome shape and increases the shape adaptability. Additionally, auxetic materials demonstrate superior energy absorption properties, which help absorb the impact of shoes on the foot when the corrective band is used as the cushioning medium to provide effective support and protect the foot.

Building on the previous discussion, it is evident that auxetic materials have widely application in orthopedics, and demonstrate the great potential as innovative biomedical solutions in orthopedics. However, certain limitations remained in some aspects of its design and manufacture; (i) the choice of filaments, wire, powder, paste, sheets, and inks, physicochemical factors, and cost remained unaddressed; (ii) thread of designed auxetic materials with a lot of small geometric features, which were not accurately manufactured due to limitations of metal 3D printing technology, particularly, the obtained porous structure differs slightly from the designed CAD model during X final hip stem model and auxetic screw production, and the strut thickness is expected to increase due to the sintering of the surrounding metal powder which adheres to the cellular structure. This mismatch would inevitably influence the auxetic mechanical properties. Therefore, the accuracy of manufacture is required to ensure the mechanical properties of the designed auxetic materials; (iii) one of the most important parameters that affect the accuracy of analytical results is the effect of considering the effective lengths of struts in a unit cell. The struts get thick in higher values of relative densities, and the overlapping of the struts at the joints should be considered in the analytical solution. The analytical solution could predict more accurate results for the actual condition of the lattice structure by defining the effective length of the struts following their orientation and thickness; (iv) the value of auxetic mechanical properties was conducted only by computational simulation, which may vary from the actual final performance. A more suitable test method was warranted to design future research; (v) experimental protocols currently lack full physiological load replication and mechanical effect consideration on surrounding tissues, bone, and diverse activities; thus, individual anatomical variations, such as medullary cavity morphology and stiffness and Poisson's ratio distribution in auxetic implants, should be considered; and (vi) these implants lack sufficient validation through animal models and clinical trials, thereby requiring further investigation for their feasibility and long‐term stability as next‐generation medical devices.

In 2016, Ding *et al*.^153^ evaluated the use of flexible absorbable polymer bone fixation devices. Absorbable bone fixation devices were made from 90% by‐weight polylactide and 10% hydroxyapatite for nonweight‐bearing bones or absorbable bone plate fixation. The study revealed that the high mechanical properties met the requirements of clinical applications. No patients required revision surgery, with no device‐related adverse events during the 6‐month follow‐up period. The fracture healing rates were 100% for both absorbable bone fixation screw and plate 6 months after implantation. Thus, auxetic materials warrant more investigation toward absorbable auxetic materials as well as experimental validation for biomedical applications before their commercialization in the biomedical sector. Recently, osteoimmunomodulation has become a new strategy for facilitating osteoimmune balance by mediating reciprocal interactions between immune and bone cells.^154^, ^155^ A recent study revealed that an osteoimmunity‐regulating DFO/MnCO@gelatin methacryloyl‐polylactide (DMGP) hybrid scaffold with biomimetically hierarchical microstructure achieved effective bone regeneration in the rat femur CSD model.^156^ Therefore, evaluating bone regenerative capacity while applying auxetic biomedical materials should be considered for orthopedic surgery, and future efforts should focus on confirming their unique biomechanical mechanisms at the cellular and molecular levels. Additionally, wearable auxetic materials with NPR were prepared by 3D printing for detecting mechanical deformation. One aspect of this is the auxetic materials that demonstrate great potential for integration with sensors. It exhibits high sensitivity and broad sensing range, emphasizing their potential applications in swallowing recognition, respiration monitoring, and joint movement detection,^157^ without affecting the potential to monitor the performance of human soft tissues and implants, and proposes new strategies for overcoming regulatory and adoption hurdles in medical devices.

## Conclusion

This review summarizes the geometric structures and models of auxetic materials, emphasizing their applications in orthopedics through AM. The materials demonstrate superior mechanical performance in various clinical orthopedic procedures, aligning well with human tissue characteristics and exhibiting promising therapeutic effects. Difficulties in NPR material application include the need for careful selection of raw materials and manufacturing methods. However, auxetic materials, with their NPR, demonstrated potential in orthopedics and surgery, particularly with AM and material science advancements.

## Ethics Statement

This study was performed in accordance with the 1964 Helsinki Declaration and was authorized by the Ethics Committee of West China Hospital.

## Conflict of Interest Statement

The authors declare that they have no competing interests or personal relationships that could have appeared to influence the work reported in this paper.

## Funding Information

This work was supported by the Sichuan Science and Technology Agency, grant number 2022NSFSC0845.

## Author Contributions

Minghao Sun, Xin Hu, Leilei Tian, Xiao Yang, Li Min prepared the manuscript; Minghao Sun, Xin Hu, Leilei Tian, Xiao Yang, Li Min revised the manuscript; and all authors read the manuscript and approved the submission.
